# Highly Porous Carbons Synthesized from Tannic Acid via a Combined Mechanochemical Salt-Templating and Mild Activation Strategy

**DOI:** 10.3390/molecules26071826

**Published:** 2021-03-24

**Authors:** Sylwia Głowniak, Barbara Szczęśniak, Jerzy Choma, Mietek Jaroniec

**Affiliations:** 1 Institute of Chemistry, Military University of Technology, 00-908 Warsaw, Poland; sylwia.glowniak@wat.edu.pl (S.G.); jerzy.choma@wat.edu.pl (J.C.); 2 Department of Chemistry and Biochemistry, Advanced Materials and Liquid Crystal Institute, Kent State University, Kent, OH 44242, USA; jaroniec@kent.edu

**Keywords:** mechanochemistry, activated carbons, non-hazardous activators, salt-templating, ball milling

## Abstract

Highly porous activated carbons were synthesized via the mechanochemical salt-templating method using both sustainable precursors and sustainable chemical activators. Tannic acid is a polyphenolic compound derived from biomass, which, together with urea, can serve as a low-cost, environmentally friendly precursor for the preparation of efficient N-doped carbons. The use of various organic and inorganic salts as activating agents afforded carbons with diverse structural and physicochemical characteristics, e.g., their specific surface areas ranged from 1190 m^2^·g^−1^ to 3060 m^2^·g^−1^. Coupling the salt-templating method and chemical activation with potassium oxalate appeared to be an efficient strategy for the synthesis of a highly porous carbon with a specific surface area of 3060 m^2^·g^−1^, a large total pore volume of 3.07 cm^3^·g^−1^ and high H_2_ and CO_2_ adsorption capacities of 13.2 mmol·g^−1^ at −196 °C and 4.7 mmol·g^−1^ at 0 °C, respectively. The most microporous carbon from the series exhibited a CO_2_ uptake capacity as high as 6.4 mmol·g^−1^ at 1 bar and 0 °C. Moreover, these samples showed exceptionally high thermal stability. Such activated carbons obtained from readily available sustainable precursors and activators are attractive for several applications in adsorption and catalysis.

## 1. Introduction

Energy demand is now greater than ever, which results in significant fossil fuel consumption and related problems, e.g., excessive emissions of greenhouse gases. Recent research efforts have been directed toward the development of new green and efficient methods for energy conversion and storage as well as the reduction of anthropogenic gaseous emissions, which are responsible for global warming effects. Most of the proposed solutions are based on the usage of supercapacitors, fuel cells and batteries that exploit porous materials. For these uses, porous carbons have attracted particular interest due to their great adsorption properties resulting from high specific surface areas (SSAs), even above 3000 m^2^·g^−1^. Activated carbons have found widespread uses for diverse applications, e.g., water purification [1], gas adsorption [2] and energy storage [3]. The great merits of these materials include widely available precursors and the simplicity of preparation. Typically, the synthesis of activated carbons consists of two steps—carbonization, in which a carbon precursor undergoes pyrolysis in an inert atmosphere, and physical or chemical activation applied to develop porosity in the carbonized material. Physical activation usually involves heating a carbonaceous material under flowing air, steam or carbon dioxide, whereas chemical activation is conducted in the presence of activating agents, e.g., KOH, H_3_PO_4_ and ZnCl_2_. Lately, an alternative salt-templating strategy has been proposed, in which eutectic salts act as both templates and activators. This method afforded hierarchical carbons that featured good pore connectivity, assuring good mass transfer compared to solely microporous carbons [4,5]. For example, Pampel et al. [4] utilized a salt-templating method to obtain porous carbons by pyrolyzing a mixture of glucose and KCl/ZnCl_2_. Varying KCl content in the mixture resulted in activated carbons with tailored porosity and high SSA in the range of 960 to 2160 m^2^·g^−1^.

Solution-based methods for the synthesis of functional materials that rely on the use of solvents have prevailed at the laboratory and industrial scales. However, these methods are usually complex, time- and energy-consuming, and contribute to the accumulation of harmful waste solutions. Therefore, the mass production of functional materials should be adjusted to meet green chemistry requirements. A field containing great promise in terms of the more environmentally friendly synthesis of porous materials is mechanochemistry [6,7]. According to the IUPAC definition, a mechanochemical reaction is a chemical reaction that is induced by direct absorption of mechanical energy during shearing, stretching, grinding, etc. [8]. The beginnings of mechanochemistry date back to prehistoric times, when early people discovered the possibility of starting a fire by rubbing two stones together. Readers are referred to the excellent reviews devoted to the history of mechanochemistry [9,10]. Mechanochemistry has been implemented in metallurgy, pharmacy, catalysis and organic chemistry [11]. Although its utilization in the fabrication of porous materials is a rather new concept, it is an up-and-coming approach to overcoming the drawbacks of solution-based synthesis methods. Nowadays, mechanochemical reactions are usually carried out in automatic ball mills, where high energy milling is available. Typically, the generated mechanical energy is directly targeted to initiate chemical conversions on the surface of solid reactants; hence, additional heating is not needed. Mechanochemistry enables researchers to conduct reactions in solvent-free conditions (neat grinding, NG) or in the presence of small amounts of solvents (liquid-assisted grinding, LAG) [6]. Recent studies have indicated that mechanochemistry can be implemented for the preparation of highly porous carbon materials from sustainable precursors and non-hazardous activators [12,13,14,15,16,17,18,19,20,21,22,23]. For example, Schneidermann et al. [12] prepared N-doped carbons with high porosity from diverse wood components—lignin (SSA of 3199 m^2^·g^−1^), tannic acid (SSA of 2873 m^2^·g^−1^), wood waste (SSA of 2988 m^2^·g^−1^), cellulose (SSA of 1870 m^2^·g^−1^) and xylan (SSA of 1163 m^2^·g^−1^). The key step in the synthetic procedure involved one-step solvent-free ball milling of carbon precursor, urea used as a nitrogen source and K_2_CO_3_ used as an activating agent. The as-prepared carbons exhibited a great performance as cathode materials for Li–S batteries with an initial discharge capacity up to 1300 mAh·g_sulfur_^−1^ (95% coulombic efficiency) and capacity retention above 75% within the first 50 cycles at a low electrolyte volume.

Theoretically, each carbon-rich material could be a precursor for activated carbons. However, to meet the demands of diverse applications, the selection of a proper precursor is very important. Biomass has shown great promise as a sustainable precursor for the preparation of functional carbons because it is a non-toxic, cheap, and renewable carbon source with carbon content even as high as 50%. Biomass employed for the preparation of activated carbons mainly originates from agriculture and forests, including dedicated crops, byproducts and residues. Producing porous carbons from biowastes is not only a cost-cutting strategy but is also a great solution for the utilization of an enormous number of presently useless products [1,2,3,24,25,26,27,28,29,30,31]. Tannins are a group of natural polyphenolic compounds extracted from various plants such as wood, fruit, bark, etc. Tannin-based extracts have already been used in various industrial sectors, e.g., as coagulants, adhesives, food additives and antioxidants [32,33]. Recently, natural tannin extracts have been proposed as efficient precursors for the synthesis of highly porous carbons [15,34,35,36] even with uniform and/or ordered porosity [5,21,37,38,39,40]. For example, Diez et al. [5] prepared carbons with SSA up to 2750 m^2^·g^−1^ using tannic acid (TA) and K_2_CO_3_/KCl mixture as salts templates. Cai et al. [15] reported the mechanochemical synthesis of porous carbons with SSA up to 1800 m^2^·g^−1^ from TA and FeCl_3_·6H_2_O. Elsewhere, implementing a salt-templating method, Tiruye et al. [21] synthesized nitrogen-functionalized porous carbon nanospheres with TA and urea used as carbon precursors. The addition of an eutectic salt of ZnCl_2_/NaCl during the synthesis led to activated carbons with SSAs up to 1570 m^2^·g^−1^. The eutectic salt mixture served as both the solvent and porogen in the synthesis. Non-carbonizable inorganic eutectic salts, which possess low melting points and are homogeneously miscible with carbon precursors, can effectively act as in situ pore forming agents, i.e., templates.

In this work, we present a green synthesis of highly porous carbons with SSAs up to 3060 m^2^·g^−1^ using a one-pot ball milling method prior to the activation step. Mechanochemical reaction of TA—an eco-friendly biomass-derived carbon precursor—in the presence of non-hazardous organic and inorganic salts afforded highly porous carbons with great gas adsorption performance. Although there are many studies in the literature devoted to mechanochemical-assisted preparations of biomass-derived carbons using chemical activators or salt-templating methods, we did not find any paper in which both strategies using organic activators were implemented. Our approach combined two methods: salt-templating and mild chemical activation using organic or inorganic salts for the preparation of micro-mesoporous carbons. The low melting point of TA ~220 °C, makes it an appropriate substrate for this templating method based on the low melting point of the eutectic salt NaCl/ZnCl_2_. Both tannic acid and the eutectic salt melt at around 200 °C–300 °C. At higher temperatures, the molten mixture undergoes carbonization and reacts with activating agents. In fact, NaCl acts as template, whereas ZnCl_2_ and the other added organic or inorganic salts, among oxalates, citrates and carbonates, support salt-templating until carbonization and then act as activating agents [4,5].

## 2. Results and Discussion

### 2.1. Morphological and Structural Characterization

**Figure 1:**
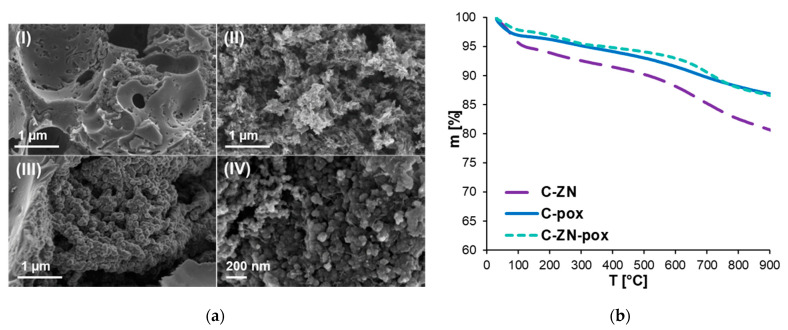
(**a**) SEM images of (**I**) C-pox, (**II**) C-ZN-pox, (**III**,**IV**) C-ZN; (**b**) thermogravimetric curves of C-ZN, C-pox and C-ZN-pox.

A series of activated carbons was prepared from tannic acid and urea in the presence of the eutectic salt NaCl/ZnCl_2_. The resulting carbons are denoted as C-ZN-*x*, where ZN refers to the eutectic salt and *x* represents the activator used—pox (potassium oxalate, K_2_C_2_O_4_), aca (ammonium carbonate, (NH_4_)_2_CO_3_), pca (potassium carbonate, K_2_CO_3_), pci (potassium citrate, K_3_C_6_H_5_O_7_) or mci (magnesium citrate, MgC_6_H_6_O_7_). For instance, C-ZN-pox refers to the carbon sample obtained from tannic acid and urea with addition of the eutectic salt NaCl/ZnCl_2_ and potassium oxalate. Furthermore, C-pox was synthesized by the same method as C-ZN-pox, but without addition of ZN. Figure 1a shows the morphology of the selected samples, C-pox, C-ZN-pox and C-ZN. The activated carbon obtained from tannic acid and urea in the presence of the eutectic salt NaCl/ZnCl_2_, i.e., C-ZN, is in the form of agglomerated spherical particles (Figure 1a(III,IV)). The morphologies of carbons activated with K_2_C_2_O_4_, namely C-pox and C-ZN-pox, differ from that of C-ZN, revealing their highly porous structures (Figure 1a(I,II)). Elemental analysis of the selected samples provides information on their element contents (Table 1). Nitrogen content ranged from 1.01 to 5.94 wt.%, even though the amount of urea used in the syntheses of all samples remained the same. The lowest nitrogen content of 1.01 wt.% was determined for the C-pox sample prepared without the addition of the eutectic salt, indicating that the presence of NaCl and ZnCl_2_ inorganic salts was favorable for high nitrogen concentration in the resulting carbons. N contents of C-ZN and C-ZN-pox were 5.60 and 5.94 wt.%, respectively. It can be noted that using an additional activator (in this case, potassium oxalate) may contribute to the high N concentration in the activated carbons. Thermogravimetric analysis performed for these selected samples indicated their high thermal stability (Figure 1b). Total mass loss varied from 19.34% to 12.90% during heating up to 900 °C in an inert atmosphere, including the mass drop at around 100 °C associated with the evaporation of adsorbed water. In the temperature range 300 °C–500 °C, a slow decomposition of the organic carbonaceous matter took place. Beyond 500 °C the observed mass loss could be mainly assigned to the elimination of heteroatoms, i.e., oxygen and nitrogen atoms. Thus, the highest weight loss was observed for the C-ZN sample with the highest content of heteroatoms (~32 wt.%) (see Table 1). The thermal stability of the prepared activated carbons is much better than other biomass-derived carbons reported in the literature [40,41].

**Figure 2:**
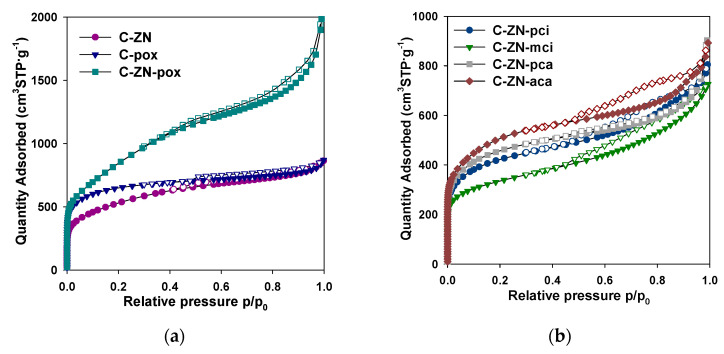
(**a**) Low-temperature nitrogen adsorption-desorption isotherms measured for C-ZN, C-pox and C-ZN-pox; (**b**) low-temperature nitrogen adsorption-desorption isotherms measured for C-ZN-aca, C-ZN-pca, C-ZN-pci and C-ZN-mci. The filled points refer to adsorption, whereas the open points correspond to desorption processes.

**Figure 3:**
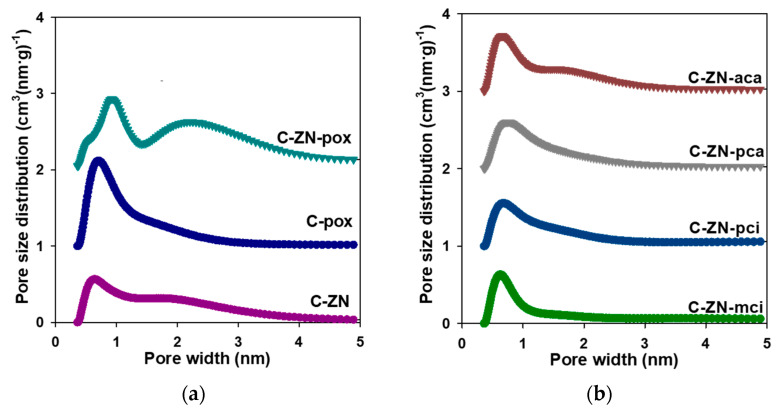
(**a**) Pore size distributions determined for C-ZN, C-pox and C-ZN-pox; (**b**) pore size distributions determined for C-ZN-aca, Z-CN-pca, C-ZN-pci and C-ZN-mci. For clarity the *y*-axis for each subsequent pore size distribution (PSD) curve was shifted upward by 1 cm^3^·(g·nm)^−1^.

Table 2 summarizes structural parameters determined from low-temperature nitrogen adsorption/desorption isotherms for all prepared samples (shown in Figure 2). The high uptake of nitrogen at low pressure indicates microporous structures, whereas the observed hystereses confirm the presence of mesopores in the obtained carbons. Therefore, the measured nitrogen isotherms are a combination of both types I and IV isotherms according to the IUPAC classification [42]. The presence of different pore sizes in the range of micro- and mesopores is also evidenced by the determined pore size distribution (PSD) functions (Figure 3) calculated in the range of micropores (<2 nm) and small mesopores (from 2 nm to 5 nm). The values of SSA and total pore volume (V_t_) ranged from 1190 to 3060 m^2^·g^−1^ and from 1.12 to 3.07 cm^3^·g^−^^1^, respectively.

In this work, the influence of various activators on the structural parameters of the resulting carbons is discussed. According to Table 1, K_2_C_2_O_4_ was the most effective agent among other salts (such as K_3_C_6_H_5_O_7_, MgC_6_H_6_O_7_, K_2_CO_3_ and (NH_4_)_2_CO_3_) used for activation of the TA-derived carbons. Using K_2_C_2_O_4_ together with the eutectic ZnCl_2_/NaCl mixture resulted in obtaining a high porosity carbon (C-ZN-pox), as evidenced by a very high SSA of 3060 m^2^·g^−1^. Its micro-mesoporous structure featured a large volume of small mesopores (up to ~4 nm), yielding high overall mesoporosity of about 75%. The reference sample C-pox, prepared analogously to C-ZN-pox except for the addition of the eutectic salt of chlorides, exhibited a smaller amount of mesopores, which contributed to its lower SSA (2330 m^2^·g^−1^). However, this sample featured the largest volume of micropores of 0.87 cm^3^·g^−1^, including a volume of ultramicropores of 0.21 cm^3^·g^−1^, among all the samples studied. It appears that both activators K_2_C_2_O_4_ and ZnCl_2_ are needed in order to develop high porosity in the TA-derived carbons with SSAs exceeding 3000 m^2^·g^−1^, which is attributed to a synergetic effect of salt-templating and chemical activation using both inorganic and organic salts [5,43]. Overall, the presence of NaCl and ZnCl_2_ salts in the synthesis generated micropores and small mesopores (between 2 and 5 nm), whereas the addition of potassium oxalate provided a large volume of micropores in the final carbons.

All the salts used in this paper acted as templates during the ball milling of carbon precursors. ZnCl_2_ and the additional potassium and ammonium salts also served as activators because they reacted with the carbon precursors upon reaching high temperatures during the one-step carbonization and activation. The templating salt, NaCl, and the residuals of other salts were simply removed by post-synthetic washing. Overall, the facile mechanochemical strategy resulted in the formation of a vast number of small pores, usually in the range of micropores and tiny mesopores (2–4 nm) in the carbon structures. The samples obtained using citrates and carbonates as activating agents featured lower values of the structural parameters SSA and V_t_ as compared to those of C-ZN-pox, C-pox and even C-ZN, which can be linked to the different condensation mechanisms during the thermal treatment process.

### 2.2. H_2_ and CO_2_ Adsorption

**Figure 4:**
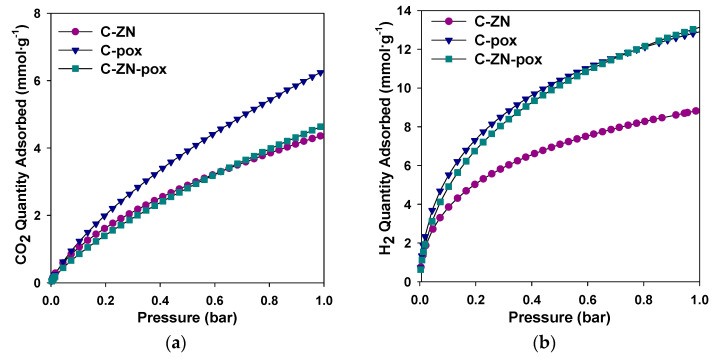
(**a**) CO_2_ adsorption isotherms measured for the selected carbons at 0 °C; (**b**) H_2_ adsorption isotherms measured for the selected carbons at −196 °C.

Carbon dioxide and hydrogen adsorption isotherms were measured for the selected samples (C-ZN, C-pox and C-ZN-pox) to further characterize their structures and adsorption properties (Figure 4). The CO_2_ and H_2_ adsorption capacities of the carbons are summarized in Table 3. As expected, C-pox adsorbed the highest amount of CO_2_—6.4 mmol·g^−1^ at 0 °C and 1 bar—because of its well-developed microporosity with a large volume of ultramicropores (V_ultra_ = 0.21 cm^3^·g^−1^). Ultramicropores provide a strong adsorption affinity toward CO_2_ molecules due to the overlapping force fields of the opposite pore walls, leading to the strong adsorption of CO_2_ molecules. Hence, the samples with similar total pore volume (C-ZN) and even the one with the highest SSA (C-ZN-pox) exhibited lower CO_2_ uptakes (4.4 and 4.7 mmol·g^−1^, respectively) due to lower volume of ultramicropores in their structures (0.13 and 0.09 cm^3^·g^−1^, respectively). These results clearly confirm that ultramicropores determine high CO_2_ uptake at ambient pressure [44]. Additionally, the prepared carbons contain nitrogen, originating from the urea used in the synthesis [12,21], which undoubtedly had an influence on their excellent CO_2_ adsorption properties, since CO_2_ molecules are attracted by N species via acid–base interactions [45,46,47]. In the case of hydrogen adsorption, the highest values of adsorption of H_2_, namely 13.2 mmol·g^−1^ and 12.9 mmol·g^−1^ at −196 °C and 1 bar, were observed for C-ZN-pox and C-pox carbons, respectively, which also showed the largest values of SSA.

Hydrogen and carbon dioxide adsorption measurements provide more comprehensive characterization of the synthesized materials and were consistent with the results obtained from the nitrogen adsorption isotherms. The simple and relatively fast mechanochemical synthesis, followed by one-step carbonization and activation presented here afforded highly porous sustainable carbons that can be used for a variety of applications such as adsorption or catalysis. For instance, the calculated structural parameters for C-ZN-pox, which revealed a high micro-mesoporous porosity, suitable for the adsorption of other gases and vapors, including volatile organic compounds as well as various pollutants from contaminated water.

Table 4 comprises the obtained results, along with reports from the literature. So far, the materials obtained in this paper belong among the most porous biomass-derived activated carbons prepared (with the highest SSAs) via the ball milling strategy [12,18,21,48,49].

## 3. Materials and Methods

### 3.1. Starting Materials

Sodium chloride (99.9%) and zinc chloride, used to prepare the eutectic mixture, were supplied by POCH S.A. Tannic acid and urea (99.9%), i.e., carbon and nitrogen precursors, were provided by the CarboSynth and Lach-Ner, respectively. Activators, namely potassium oxalate (99.5%), potassium carbonate (99%) and ammonium carbonate, were purchased from CHEMPUR (Piekary Śląskie, Poland), whereas potassium citrate (99%) was from Sigma-Aldrich (Munich, Germany) and magnesium citrate (95%) was from Fluka (Munich, Germany). All chemicals were used without further purification.

### 3.2. Synthesis Procedure

**Figure 5:**
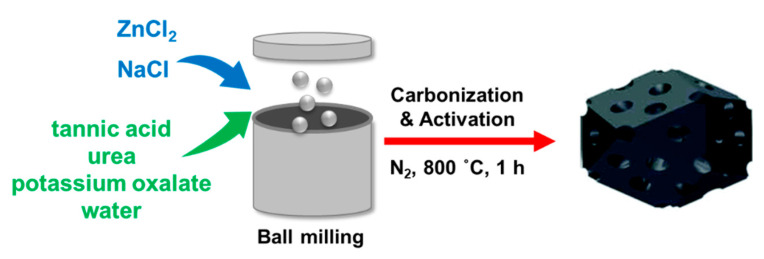
Scheme illustrating the synthesis of tannic acid-derived activated carbons.

To prepare highly porous carbons we employed and modified the facile mechanochemical concept for the synthesis of porous carbons from tannic acid (TA) and urea (U) reported by Tiruye et al. [21]. Herein, we used additional/different salts to develop the porosity of the resulting activated carbons. In brief, a eutectic salt was prepared by 5 min milling of ZnCl_2_ and NaCl with a 29% mole fraction of NaCl in Planetary Mono Mill Pulverisette classic line ball milling machine (Fritsch, Bahnhofstraße, Germany). Then, TA and U were added, together with one of the following activators—K_2_C_2_O_4_, K_3_C_6_H_5_O_7_, MgC_6_H_6_O_7_, K_2_CO_3_ or (NH_4_)_2_CO_3_—and small amount of deionized water (adjusted to obtain a semi-solid homogenous paste) and mixed in the ball miller for 1 h at 500 rpm using a molar ratio of 13:1 (U:TA). The mass ratio of precursors (TA + U) to the eutectic salt (ZnCl_2_/NaCl) was 1:3, whereas the mass ratio of the additional salt to the eutectic salt was 1:1. The as-prepared paste was activated at 800 °C in nitrogen atmosphere for 1 h. Next, the resulting solid was purified in 1 M HCl and stirred with a magnetic stirrer overnight to remove residues from pores. Finally, it was washed several times with deionized water, filtered and dried overnight in an oven at 80 °C. The as-prepared carbons are denoted as C-ZN-*x*, where ZN refers to the eutectic salt and *x* represents the activator used—pox (potassium oxalate, K_2_C_2_O_4_), aca (ammonium carbonate, (NH_4_)_2_CO_3_), pca (potassium carbonate, K_2_CO_3_), pci (potassium citrate, K_3_C_6_H_5_O_7_) or mci (magnesium citrate, MgC_6_H_6_O_7_). Additionally, C-pox was obtained using the same recipe as C-ZN-pox, except for the addition of ZN. Figure 5 shows a schematic representation of the synthesis procedure.

### 3.3. Measurements and Calculations

The SEM images were performed by using a scanning electron microscope LEO 1530 manufactured by Zeiss (Oberkochen, Germany) at 2 kV acceleration voltage. The thermal stability of activated carbons was examined by thermogravimetric analysis performed on Setaram Labsys TG (Caluire-et-Cuire, France) under the following operational conditions: heating rate 5 °C·min^−1^, nitrogen atmosphere, temperature range of 30 °C–900 °C. An elemental analysis to assess C, H and N wt.% content was performed using a Vario EL Cube apparatus (Elementar, Langenselbold, Germany). Nitrogen adsorption isotherms were measured at −196 °C using a ASAP 2020 volumetric analyzer manufactured by Micromeritics Instrument Corp. (Norcross, GA, USA). Hydrogen and carbon dioxide isotherms were measured at −196 °C and 0 °C, respectively. All samples were outgassed for 2 h at 200 °C prior to the adsorption measurements. Specific surface area (SSA) was calculated from low temperature nitrogen adsorption data in a relative pressure range of 0.05 < *p*/*p*_0_ < 0.2 using the Brunauer–Emmett–Teller method [50]. Total pore volume (V_t_) was calculated using the volume of liquid nitrogen adsorbed at a relative pressure ~0.99. Pore size distribution (PSD) and micropore volume (V_micro_) were calculated from low-temperature nitrogen adsorption data using the non-local density functional theory (2D-NLDFT) method for carbons with slit-shaped pores, taking into account the energetic heterogeneity and geometrical corrugation of the surface [51,52]. The calculations were performed using the numerical program SAIEUS. The mesopore volume (V_meso_) was calculated as the difference between V_t_ and V_micro_.

## 4. Conclusions

This work demonstrates the mechanochemical synthesis of N-doped activated carbons from sustainable precursors tannic acid and urea using a combined salt-templating strategy and chemical activation. This mechanochemical approach is environmentally friendly due to the reduction of both synthesis time and solvent usage compared to more traditional wet chemistry. Moreover, the biomass-derived compound tannic acid is employed as a carbon source and sustainable inorganic and organic salts are used as templates and activators. To show the influence of different activators on the resulting carbons, a series of organic and inorganic salts was examined—K_2_C_2_O_4_, K_3_C_6_H_5_O_7_, MgC_6_H_6_O_7_, K_2_CO_3_ and (NH_4_)_2_CO_3_. Their physicochemical characterization revealed that the activators used had a significant impact on the morphology, thermal stability, porosity and adsorption properties of the obtained carbons. In particular, simultaneous activation by zinc chloride and potassium oxalate led to an outstanding porosity in the resulting carbon (SSA of 3060 m^2^·g^−1^ and V_t_ of 3.07 cm^3^·g^−1^) and superior gas adsorption properties (H_2_ and CO_2_ uptakes at ambient pressure, −196 and 0 °C, equal to 13.2 mmol·g^−1^ and 4.7 mmol·g^−1^, respectively). The presented mechanochemical salt-templating method complies with the principles of Green Chemistry and is a promising method for the large-scale production of highly porous activated carbons. It should be emphasized that although the used mechanochemical route is green, a purification step involving washing with HCl (an acidifying reagent for the environment) is often needed, as in this case. Moreover, zinc-based salts are also not recommended as activation agents, due to the related environmental problems. In our approach, the ZnCl_2_ content in the synthesis of most activated carbons was about ~36 wt.%, except for the reference sample, C-ZN (~63 wt.%). Given the above, we also prepared activated carbon without the addition of Zn-containing salts and therefore without the necessity of using HCl solution for washing. It was revealed that using only three sustainable carbon-rich reagents—tannic acid, urea and potassium oxalate—afforded a micro-mesoporous carbon with a high SSA of 2330 m^2^·g^−1^ and a CO_2_ uptake capacity as high as 6.4 mmol·g^−1^ at 1 bar, which makes it an attractive sorbent for real applications.

## Figures and Tables

**Table 1:** Element concentrations in the selected activated carbon samples.

Samples	C (wt.%)	H (wt.%)	N (wt.%)
C-ZN	67.52	2.84	5.60
C-pox	71.02	2.19	1.01
C-ZN-pox	74.20	1.83	5.94

**Table 2:** Structural parameters calculated from low-temperature (−196 °C) nitrogen adsorption data for all samples studied.

Sample	SSA ^1^ (m^2^·g^−1^)	V_t_^2^ (cm^3^·g^−1^)	V_ultra_^3^ (cm^3^·g^−1^)	V_micro_^4^ (cm^3^·g^−1^)	V_meso_^5^ (cm^3^·g^−1^)
C-ZN	1910	1.34	0.13	0.60	0.74
C-pox	2330	1.35	0.21	0.87	0.48
C-ZN-pox	3060	3.07	0.09	0.77	2.30
C-ZN-aca	1832	1.38	0.16	0.61	0.77
C-ZN-pca	1645	1.40	0.11	0.56	0.84
C-ZN-pci	1520	1.25	0.12	0.50	0.75
C-ZN-mci	1190	1.12	0.14	0.38	0.74

^1^ SSA—specific surface area, calculated by the Brunauer–Emmett–Teller method. ^2^ V_t_—total pore (single-point) volume, obtained from the amount of adsorbed nitrogen at *p*/*p*_0_ ≈ 0.99. ^3^ V_ultra_—volume of ultramicropores (pores <0.7 nm). ^4^ V_micro_—volume of micropores (pores <2.0 nm). ^5^ V_meso_—volume of mesopores–difference between V_t_ and V_micro_.

**Table 3:** Carbon dioxide and hydrogen uptakes for the selected carbon samples.

Samples	CO_2_ (mmol·g^−1^) 0 °C, 1 bar	H_2_ (mmol·g^−1^) −196 °C, 1 bar
C-ZN	4.4	8.9
C-pox	6.4	12.9
C-ZN-pox	4.7	13.2

**Table 4:** Comparison of biomass-derived activated carbons obtained via mechanochemically-assisted syntheses.

Sample	Carbon Source/Activator	SSA [m^2^·g^−1^]	Application	Ref.
C-ZN	Tannic acid/ZnCl_2_	1910	CO_2_ adsorption (4.4 mmol·g^−1^ at 0 °C and 1 bar)	This work
			H_2_ adsorption (8.9 mmol·g^−1^ at −196 °C and 1 bar)	
C-pox	Tannic acid/K_2_C_2_O_4_	2330	CO_2_ adsorption (6.4 mmol·g^−1^ at 0 °C and 1 bar)	This work
			H_2_ adsorption (12.9 mmol·g^−1^ at −196 °C and 1 bar)	
C-ZN-pox	Tannic acid/ZnCl_2_, K_2_C_2_O_4_	3060	CO_2_ adsorption (4.7 mmol·g^−1^ at 0 °C and 1 bar)	This work
			H_2_ adsorption (13.2 mmol·g^−1^ at −196 °C and 1 bar)	
LUPC	Lignin/K_2_CO_3_	3199	Li–S batteries	[12]
WWUPC	Wood waste/K_2_CO_3_	2988	Li–S batteries	[12]
TUPC	Tannic acid/K_2_CO_3_	2873	Li–S batteries	[12]
LD2600P	Lignin/KOH	2224	CO_2_ adsorption (4.5 mmol·g^−1^ at 25 °C and 1 bar)	[48]
NDAB3-500	*Arundo donax* and chitosan/ZnCl_2_	1863	CO_2_ adsorption (2.1 mmol·g^−1^ at 25 °C and 1 bar)	[18]
HSAC-MCS-900-9	Coconut shel/-	1771	Li–S batteries and creatinine adsorption	[49]
TA_0	Tannic acid/ZnCl_2_	1570	Supercapacitors	[21]
SD2650P	Sawdust/KOH	1313	CO_2_ adsorption (5.8 mmol·g^−1^ at 25 °C and 1 bar)	[48]

## Data Availability

Data are available from the authors on request.

## Sample Availability

Carbon samples are available from the authors.
